# Association between Genetic Polymorphisms of *miR-1307*, *miR-1269*, *miR-3117* and Breast Cancer Risk in a Sample of South East Iranian Women

**DOI:** 10.31557/APJCP.2021.22.1.201

**Published:** 2021-01

**Authors:** Sahel Sarabandi, Hedieh Sattarifard, Mohammad Kiumarsi, Shima Karami, Mohsen Taheri, Mohammad Hashemi, Gholamreza Bahari, Saeid Ghavami

**Affiliations:** 1 *Department of Clinical Biochemistry, School of Medicine, Zahedan University of Medical Sciences, Zahedan, Iran. *; 2 *Department of Human Anatomy and Cell Science, Max Rady College of Medicine, Rady Faculty of Health Sciences, University of Manitoba, Winnipeg, MB R3E 3P4, Canada. *; 3 *Genetics of Non-Communicable Disease Research Center, Zahedan University of Medical Sciences, Zahedan, Iran. *; 4 *Children and Adolescent Health Research Center, Resistant Tuberculosis Institute, Zahedan University of Medical Sciences, Zahedan, Iran. *; 5 *Faculty of Medicine, Katowice School of Technology, 40-555 Katowice, Poland. *; 6 *Autophagy Research Center, Faculty of Medicine, Shiraz University of Medical Sciences, Shiraz, Iran. *

**Keywords:** Breast cancer, microRNA, polymorphism, cancer susceptibility

## Abstract

**Introduction:**

MicroRNAs (miRNAs) play an essential role in the susceptibility and development of cancer cells.

**Objective:**

Examining the dependency of breast cancer risk with genetic polymorphisms of* miR-1307, miR-1269*, and *miR-3117* in a sample of Iranian women (southeast region).

**Methods:**

The case-control study consisted of 520 individuals (260 diagnosed BC patients, 260 healthy individuals). The polymerase chain reaction-restriction fragment length polymorphism (PCR-RFLP) method was used for genotyping of *miR-1307 *rs7911488*, miR-1269* rs73239138*,* and *miR-3117* (rs4655646 and rs7512692) polymorphisms.

**Results and Conclusion:**

This study provided evidence that *miR-1307* rs7911488 polymorphism significantly reduced the risk of BC in heterozygous AG genotype, as well as dominant (AG+GG) genotype and G allele. A significant correlation was found between dominant (AA+AG) genotype, the A allele and protection against BC due to *miR-1269* rs73239138 in the sample of study. In contrast, our findings suggested that AG genotype and G allele of *miR-3117* rs4655646 polymorphism could increase BC’s susceptibility among the southeastern Iranian females. The *miR-3117* rs7512692 variant also increased the risk of BC in codominant, dominant and recessive models, as well as the T allele. The possible dependency of *miR-1307, miR-1269*, and* miR-3117* variants with patients’ clinicopathological characteristics and BC was also studied. It was concluded that there is a correlation between *miR-3117* rs7512692 variant and tumor grade (p=0.031); also, a correlation between *miR-1269* rs73239138 variant and progesterone receptor status (p=0.006). The current investigation revealed that *miR-1307, miR-1269*, and *miR-3117* polymorphisms might play a crucial role in the Iranian population’s vulnerability to BC.

## Introduction

Breast cancer (BC), one of the most frequent carcinomas amongst women, is the second leading cancer-related cause of death in women worldwide (Bray et al., 2018). Breast cancer may occur in several parts of the breast, including lobules, ducts, and connective tissue. However, it commonly occurs in the inner lining of milk ducts or the lobules which produce milk. Out of control cell division in these tissues could invade other breast tissues and move to the lymph nodes and cause metastasis to other organs (Winer, 2001; Yu, 2019; Umami et al., 2020), including bones, lung, liver, and brain (Eckhardt et al., 2012; Vickers, 2017).

The breast cancer incidence rate ranges widely all over the world. According to GLOBOCAN 2018 report, the global average BC incidence rate was 46.3 per 100000 individuals, while Australia and New Zealand reported the highest rate (94.2) and South-Central Asia recorded the lowest rates (25.9) (Bray et al., 2018). Based on GLOBOCAN 2018, the Iranian BC incidence rate was 31.0 per 100,000 (Bray et al., 2018).

Screening of numerous BC patients demonstrated that the risk of BC might be associated with several factors, including genetic mutation, family history, lifestyle, alcohol consumption, level of estrogen and progesterone hormones, and physical activity (Jayasekara et al., 2016; Pizot et al., 2016; Dall and Britt, 2017; Shiyanbola et al., 2017; Bertoni et al., 2019; McTiernan et al., 2019). Numerous genetic mutations might occur in the human genome, responsible for the permanent alternation in DNA or RNA sequences. A Single Nucleotide Polymorphism (SNP) is a replacement of a nucleotide at several positions in the human genome (Kitts and Sherry, 2002). Studies by Dr. Hashemi’s research team showed that there had been relationships between SNPs and different diseases, including breast cancer (Hassanzarei et al., 2017), prostate cancer (Hassanzarei et al., 2017; Sattarifard et al., 2019), lung cancer (Moazeni-Roodi et al., 2019), colorectal cancer (Hashemi et al., 2018), bladder cancer (Sadeghi-Bojd et al., 2019), nephrotic syndrome (Sadeghi-Bojd et al., 2019), nonsyndromic cleft lip (Rafighdoost et al., 2015; Rafighdoost et al., 2017; Rafighdoost et al., 2018; Rafighdoost et al., 2019), and nonalcoholic fatty liver disease (Hashemi et al., 2013).

The transcriptome of genome and tiling array studies provided evidence that over 90 percent of humans’ genomic DNA is not translated to any proteins (Non-coding RNAs), and only two percent is expected to translate into functional proteins (Rinn and Chang, 2012). According to the original transcription size, non-coding RNAs are clustered into two main groups, the small Non-Coding RNAs (shorter than 200 nucleotides), including miRNAs and snoRNAs piwiRNAs, and siRNAs; and long non-coding RNAs which contain more than 200 nucleotides (Bhan and Mandal, 2015). It is shown that miRNAs have multiple functions, including gene regulation. Matured miRNA could bind to the 3′-untranslated section (3′UTR) of specific mRNAs, which might lead to regulating gene expression (Meijer et al., 2013; Peng and Croce, 2016). miRNAs are key regulators of the human’s transcriptome and function as oncogenes or tumor suppressor genes depending on their target genes’ function. Recent evidence showed that genetic variations in miRNA genes could affect the expression, biogenesis of miRNA, or target selection, which in turn affects their target genes’ expression and the cancer progress (Hu et al., 2008; Omrani et al., 2014, Liu et al., 2016; Wang et al., 2016; Sibin et al., 2017; Wu et al., 2018).

*miR-1307-3p *was recently recognized as a cancer-related miRNA. The studies showed that miR-1307-3p could be involved in critical biological pathways, including proliferation, differentiation, lymphocytes activation, nucleotide synthesis, and metabolism (Zhou et al., 2015; Qiu and Dou, 2017; Yang et al., 2018). The relation between miR-1307-3p and cancers, including renal cell carcinoma (RCC) (García-Donas et al., 2016), ovarian cancer (Zhou et al., 2015), colorectal cancer (Tang et al., 2015) and breast cancer (Shimomura et al., 2016) has been previously reported. Furthermore, recent BC research showed that the elevated level of *miR-1307-3p *in serum could be used to identify the BC patients in their early stages (Shimomura et al., 2016). *miR-1269 *is located at human chromosome 4, and recent studies suggested that it could act as an oncomir (ono miRNA) (Yang et al., 2014; Bu et al., 2015). The correlation between *miR-1269* and prostate cancer metastasis and progression of primary hepatocellular carcinoma (HCC) has been approved in recent studies (Yang et al., 2014; Bu et al., 2015). Despite all these documents, other investigations show the controversial function of mir-1269 as a tumor suppressor gene in gastric cancer (Li et al., 2017) and HCC (Xion et al., 2015).* miR-3,117* is located at human chromosome 1. The significant association between mir-3117 and many types of cancers, including colorectal cancer (Neerincx et al., 2015), HCC (Cui et al., 2017), and acute lymphoblastic leukemia (Gutierrez-Camino et al., 2018) was recently examined. No studies have investigated the susceptibility of BC risk with *miR-3117* though.

In the current this investigation, the correlation between *miR-3117* rs4655646, *miR-3117* rs7512692, *miR-1269* rs73239138, *miR-1307-3p* rs7911488 and the risk of BC in a sample of southeast Iranian women was evaluated. 

## Materials and Methods

*Patients*

Our population-based case-control study consists of 520 individuals, including 260 histologically confirmed BC patients and 260 age-matched population-based healthy women

with no history of any type of cancer and (samples are not related to the patients of the study). The protocol of this study has been designed based on previous investigations (Danesh et al., 2018; Hashemi et al., 2018; Karami et al., 2020). The Institutional Review Board approved this study to be conducted at the Zahedan University of Medical Sciences (IR.ZAUMS.REC.1397.385). Proper consent was obtained from all participants. The genomic DNA samples were extracted using the salting-out technique and were collected in separate special tubes containing EDTA (Hashemi et al., 2013).

*Genotyping*

**Figure 1:**
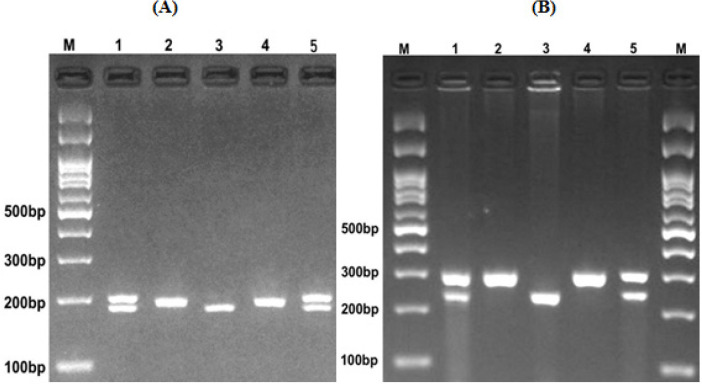
Electrophoresis Pattern of: (A) miR-1307 rs7911488 (A>G) polymorphism. M: DNA marker; Lanes 1, 5, AG; Lanes 2, 4, AA; Lane 3, GG. (B) Electrophoresis pattern of miR-1269 rs73239138 (A/G) polymorphism. M, DNA marker; Lanes 1, 5, AG; Lanes 2, 4, GG; Lane 3, AA. M: DNA marker

**Figure 2:**
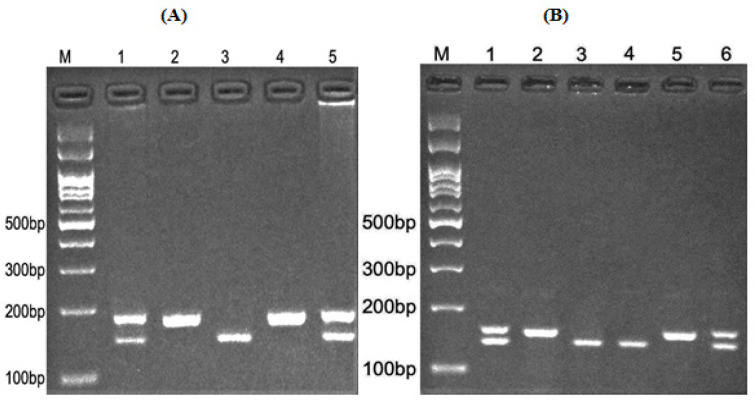
Electrophoresis Pattern of, (A) miR-3117 rs4655646 (A/G) polymorphism; M, DNA marker; Lanes 1, 5, AG; Lanes 2, 4, AA; Lane 3, GG. (B) Electrophoresis pattern of miR-3117 rs7512692 (C/T) polymorphism. M, DNA marker; Lanes 1, 6, TC; Lanes 2, 5, TT; Lanes 3, 4, CC

Polymerase Chain Reaction-Restriction Fragment Length Polymorphism (PCR-RFLP) was used to genotype *miR-1269, miR-1307*, and *miR-3117* genes polymorphisms. The protocol used for this research, previously used by Dr. Hashemi’s lab, is as follow (Hassanzarei et al., 2017; Payehghadr et al., 2018; Nazarian et al., 2019):

1. The volume for assembling 17 µl of the reaction solution in each PCR tube is presented in Table 1.

2. The sequences of primers used for detection of *miR-1307, miR-1269* and *miR-3117* polymorphisms are listed in Table 2.

3. PCR thermal cycling conditions, which were used for amplification of *miR-1307, miR-1269 *and *miR-3117 *polymorphisms, are listed in Table 3.

4. PCR products were digested with corresponding restriction endonucleases.

5. In the last pace, a UV transilluminator was used to detect and visualize the digested fragments, which were separated by agarose gel electrophoresis. Briefly, for miR-1307 rs7911488, the HhaI restriction enzyme digested the G allele and produced 18 and 176 base pair (bp) pattern, while the A allele was undigested (194 bp fragment) (Figure 1A), miR-1269 rs73239138 A allele was digested by BstXI restriction enzyme and produced a 58 and 237 bp fragments, while the G allele was undigested (295 bp fragment) (Figure 1B), miR-3117 rs4655646 G allele was digested by TaqI restriction enzyme and produced 39 and 143 bp fragments, while A allele was undigested (182 bp fragment) (Figure 2A), and Hpy1888III restriction enzyme digested miR-3117 rs7512692 C allele and produced 21 and 136 bp fragments, while T allele was undigested (157 bp fragment) (Figure 2B). 

*Statistical analysis*

Statistical analyses were done using SPSS 22 software. To estimate the Hardy–Weinberg equilibrium (HWE) among the controls, the* χ*^2^ test was used. The correlation between the genotypes and risk of BC was measured by odds ratios (ORs) with 95% confidence intervals (Erdmann, Szymanski et al.). Unconditional logistic regression analysis was applied to assess the relationship between *miR-1269, miR-1307* and *miR-3117* genes polymorphisms and BC risk. The p-value of less than 0.05 was considered statistically significant.

## Results

The present study included 520 participants. The study consisted of 260 females who were diagnosed with breast cancer with an age group of 48.09 ±10.59 and 260 healthy females with an age group of 46.26±10.72 recruited in the study. There was not a significant age difference between the patient and control groups (p=0.052). Frequency of alleles and genotyping of *miR-1307*, rs7911488, *miR-1269, rs73239138, miR-3117*, rs4655646 and rs7512692 polymorphisms among the study group and the control group are presented in Table 4./

Our findings show that miR-1307 rs7911488 polymorphism has significantly reduced the risk of BC in heterozygous genotype AG (OR=0.28, 95%CI=0.19-0.40, P<0.001, A/G vs A/A), dominant (OR=0.30, 95%CI=0.21-0.43, P<0.001, A/G+G/G vs A/A), and G allele (OR=0.50, 95%CI=0.38-0.67, P<0.001, G vs A). Similarly, the *miR-1269* rs73239138 G>A polymorphism significantly decreased the risk of BC in dominant (OR=0.64, 95%CI=0.42-0.98, P=0.048, A/A+A/G vs G/G), and A allele (OR=0.66, 95%CI=0.45-0.93, p=0.041, A vs G). Our investigation also showed that *miR-3117 *rs4655646 is a risk factor for BC in heterozygous genotype AG (OR=1.93, 95%CI=1.05-3.51, P=0.030, A/G vs A/A), and G allele (OR=2.08, 95%CI=1.20-3.62, p=0.014, G vs A). So as *miR-3117* rs7512692 C>T polymorphism increased the risk of BC in C/T heterozygous genotypes (OR=4.58, 95%CI=2.87-7.44, p<0.001, C/T vs C/C) and T/T homozygous (OR=12.80, 95%CI=6.72-24.12, p<0.001, T/T vs C/C), dominant (OR=5.70, 95%CI=3.58-9.06, p<0.001, C/T+T/T vs C/C), recessive (OR=4.27, 95%CI=2.55-7.22, p<0.001, T/T vs C/C+C/T) and T allele (OR=2.75, 95%CI=2.14-3.54, p<0.001, T vs C) genetic models.

Furthermore, the correlation between the variants and clinicopathological characteristics, including age, size of the tumor, lymph node, histology, tumor grade, the status of estrogen and progesterone receptors was examined. Our results showed that there was a significant association between *miR3117* rs7512692 C>T and tumor grade (p=0.031) and *miR-1269* rs73239138 G>A with progesterone receptor status (p=0.006) Table 5.

**Table 1:** The Volumes for Assembling the Reaction Solution in Each PCR Tube for Detection of miR-1307, miR-1269 and miR-3117 Polymorphisms

Polymorphisms	Reverse Primer	Forward Primer	2X Taq master mix	H_2_O	DNA
miR-1307 rs7911488	1 µl	1 µl	8 µl	6 µl	1 µl
miR-1269 rs73239138	1 µl	1 µl	8 µl	6 µl	1 µl
miR-3117 rs4655646	1 µl	1 µl	8 µl	6 µl	1 µl
miR-3117 rs7512692	1 µl	1 µl	8 µl	6 µl	1 µl

**Table 2:** The Sequences of Primers Used for Detection of miR-1307, miR-1269 and miR-3117 Polymorphisms

Polymorphisms	Primer sequence (5`=>3`)	Restriction Enzyme	Fragment (bp)
miR-1307	F: TCTGGAAGAATATATAGCAAAGGCAGCTT	HhaI	Allele A=194 bp
rs7911488	R: CTCGACCGGCTCGTCTGCG		Allele G=176+18 bp
miR-1269	F: ACAAACTATTGCTCTCTTTCTTGCTT	BstXI	Allele G=295 bp
rs73239138	R: GGAGGCTGAGAAGTCTCATGATA		Allele A= 237+58 bp
miR-3117	F: TGGCATGTGAGGAAAGTTGGA	TaqI	Allele A=182 bp
rs4655646	R:AGATATTGGGCCTCTACCCGT		Allele G=143+39 bp
miR-3117	F: TGGCAGTTGCTGGTACTCTT	Hpy188III	Allele T=157 bp
rs7512692	R: CTCAAGTCTCCTCCCCCATC		Allele C=136+21 bp

**Table 3:** PCR Thermal Cycling Conditions for Amplification of miR-1307,miR-1269 and miR-3117 Polymorphism

Polymorphism	Denaturation		Annealing		Extension		Cycles
	Time	Temp	Time	Temp	Time	Temp	
miR-1307 rs7911488	30s	95^0^C	30s	58^0^C	30s	72^0^C	30
miR-1269 rs73239138	30s	95^0^C	30s	62^0^C	30s	72^0^C	30
miR-3117 rs4655646	30s	95^0^C	30s	60^0^C	30s	72^0^C	30
miR-3117 rs7512692	30s	95^0^C	30s	58^0^C	25s	72^0^C	30

**Table 4:** The Association of miR-1307, miR-1269 and miR-3117 Polymorphisms and Breast Cancer Risk

Polymorphism	Case n (%)	Control n (%)	OR (95%CI)	p
miR-1307 rs7911488				
Codominant				
AA	155 (59.6)	80 (30.8)	1	-
AG	90 (34.6)	167 (64.2)	0.28 (0.19-0.40)	<0.001
GG	15 (5.8)	13 (5)	0.59 (0.28-1.30)	0.213
Dominant				
AA	155 (59.6)	80 (30.8)	1	-
AG+GG	105 (40.4)	180 (69.2)	0.30 (0.21-0.43)	<0.001
Recessive				
AA+AG	245 (94.2)	247 (95)	1	-
GG	15 (5.8)	13 (5)	1.16 (0.53-2.41)	0.846
Allele				
A	400 (76.92)	327 (62.88)	1	-
G	120 (23.08)	193 (37.12)	0.50 (0.38-0.67)	<0.001
miR-1269 rs73239138				
Codominant				
GG	216 (83.1)	197 (75.8)	1	-
GA	39 (15)	55 (21.2)	0.65 (0.41-1.01)	0.067
AA	5 (1.9)	8 (3.0)	0.57 (0.21-1.82)	0.403
Dominant				
GG	216 (83.1)	197 (75.8)	1	-
AA+AG	44 (16.9)	63 (24.2)	0.64 (0.42-0.98)	0.048
Recessive				
GG+AG	255 (98.1)	252 (96.9)	1	-
AA	5 (1.9)	8 (3.1)	0.62 (0.19-1.91)	0.576
Allele				
G	471 (90.58)	449 (86.35)	1	-
A	49 (9.42)	71 (13.65)	0.66 (0.45-0.93)	0.041
miR-3117 rs4655646				
Codominant				
AA	224 (86.1)	241 (92.7)	1	-
AG	34 (13.1)	19 (7.3)	1.93 (1.05-3.51)	0.03
GG	2 (0.8)	0	-	-
Allele				
A	482 (92.69)	501 (96.35)	1	-
G	38 (7.31)	19 (3.65)	2.08 (1.20-3.62)	0.014
rs7512692 Codominant				
CC	28 (10.8)	106 (40.8)	1	-
CT	161 (61.9)	133 (51.2)	4.58 (2.87-7.44)	<0.001
TT	71 (27.3)	21 (8.0)	12.80 (6.72-24.12)	<0.001
Dominant				
CC	28 (10.8)	106 (40.8)	1	-
CT+TT	232 (89.2)	154 (59.2)	5.70 (3.58-9.06)	<0.001
Recessive				
CC+CT	189 (72.7)	239 (91.9)	1	-
TT	71 (27.3)	21 (8.1)	4.27 (2.55-7.22)	<0.001
Allele				
C	217 (41.73)	345 (66.35)	1	-
T	303 (58.27)	175 (33.65)	2.75 (2.14-3.54)	<0.001

**Table 5.** The Association of miR-1307, miR-1269 and miR-3117 Polymorphisms with Clinicopathological Characteristics of Breast Cancer (BC) Patients

Characteristic of patients	miR-1307 rs7911488			P-value	miR-1269 rs73239138			P-value	miR-3117 rs4655646			P-value	miR-3117 rs7512692			P-value
	AA	AG	GG		GG	GA	AA		AA	AG	GG		CC	CT	TT	
Age, years				0.731				0.63				0.766				0.835
≤50	91	55	7		127	22	4		130	22	1		18	94	41	
>50	63	35	7		88	16	1		92	12	1		10	65	70	
Tumor size, cm				0.133				0.991				0.709				0.743
≤2	34	17	0		42	8	1		46	5	0		7	30	14	
>2	100	66	11		145	28	4		155	20	2		18	112	47	
Histology				0.924				0.064				0.694				0.057
Ductal carcinoma	126	74	12		180	29	3		187	23	2		28	123	61	
Others	19	13	2		24	8	2		29	5	0		0	25	9	
Lymph node metastasis				0.817				0.619				0.362				0.123
No	75	43	8		107	17	2		107	18	1		10	75	41	
Yes	56	37	5		79	16	3		89	8	1		14	62	22	
Grade				0.416				0.094				0.57				0.031
I	16	12	51		22	10	0		27	5	0		3	25	4	
II	66	36	34		93	12	2		94	11	2		10	57	40	
III+IV	51	5	4		73	14	2		78	11	0		14	53	22	
Stage				0.155				0.368				0.67				0.299
I	9	5	0		13	1	0		13	1	0		0	7	7	
II	69	38	3		87	20	3		96	14	0		11	70	29	
III	36	25	5		56	10	0		58	7	1		8	43	15	
IV	16	11	5		27	3	2		26	6	0		5	16	11	
Estrogen receptor status				0.556				0.082				0.593				0.655
Positive	99	54	7		133	26	1		139	19	2		18	100	42	
Negative	47	30	6		68	11	4		73	10	0		10	47	26	
Progesterone receptor status				0.834				0.006				0.187				0.082
Positive	94	51	8		126	27	0		129	22	2		133	26	1	
Negative	50	32	5		72	10	5		80	7	0		68	11	4	
HER2 status				0.392				0.371				0.279				0.562
Positive	43	29	6		68	8	2		66	12	0		8	51	19	
Negative	103	55	7		134	28	3		147	16	2		20	96	49	

## Discussion

Recent investigations showed that miRNAs are involved in regulating more than 30% of the human genome (Filipowicz et al., 2008). It is suggested that miRNAs may be involved in many biological pathways, including proliferation and metastasis. However, the primary function of miRNAs has not been identified yet (Bueno et al., 2008). Several studies have shown that there is a strong correlation between abnormal expression of miRNAs and risk of BC (Qi et al., 2015; Danesh et al., 2018; Moazeni-Roodi et al., 2019). Recently, Several investigations have been conducted on *miR-1307, miR-1269,* and *miR-3117* polymorphisms to clarify the direct association of these variants in cancer susceptibility.

The association of microRNA’s such as *miR-1307, miR-1269,* and *miR-3117* polymorphisms with risk of cancer has been demonstrated (Gan et al., 2015; Xiong et al., 2015; Cui et al., 2017; Bao et al., 2018; Wang and Zhu, 2018; Han et al., 2019).

The current investigation In this study, for the first time in Iran, has examined the possible association between miR-1307 rs7911488, miR-1269 rs73239138, miR-3117, rs4655646, rs7512692 and the risk of BC in a sample of women in southeast of Iran was studied.

Current findings proposed that the AG genotype, as well as the G allele of rs7911488 of miR-1307 polymorphism, significantly reduced the risk of BC among the sample group of southeast Iranian women. Only two studies have already investigated the impact of rs7911488 miR-1307 on cancer so far. For the first time, Tang et al., (2015) showed that *miR-1307* is involved in the overexpression of Bcl2 and increasing the risk of colorectal cancer, which is contradictory to our results. The contradiction between these two findings could be due to the different ethnicities of samples and cancer types being studied. 

Furthermore, Qi et al., (2017) showed that *miR-1307* polymorphism is involved in capecitabine-based chemotherapy in patients who were diagnosed with colon cancer. Their results showed that the response rate of capecitabine-based treatment in the patients with TC genotype was the highest while CC genotype had the lowest chemotherapy response (Min et al., 2017).* miR-1269* was identified as an ono miRNA, which could act as a tumor suppressor (Bu et al., 2015). miR-1269 is located at chromosome 4. So far, only three studies have examined the relation between *miR-1269* rs73239138 and vulnerability to cancer. In 2015, Guanying et al., (2015) observed that the AG and AA genotype of *miR-1269* rs732- 39138 decreased the risk of susceptibility to HCC in the southern Chinese population (Xiong et al., 2015). In contrast to the previous findings, Pei Min et al. showed that *miR-1269* rs73239138 considerably increased the risk of HCC in the eastern Chinese population (Min et al., 2017). In 2017, the results of Wenshual Li et al. showed that the downregulating of Zinc Finger Protein70 (ZNF70) by *miR-1269 *variant rs73239138 was a protective factor against gastric cancer (Li et al., 2017). Our current results suggest that there is a significant correlation between dominant (AA+AG) genotype, the A allele and protection against BC due to *miR-1269 *rs73239138 in the Iranian population. 

It is important to note that the study of the correlation between polymorphisms in a specific type of cancer and a certain genotype is heavily dependent on the type of cancer as well as and the ethnic population under investigation. This may cause possible contradictions among different research results. 

miR-3117 is located at human chromosome 1. The association between the development and metastasis of colorectal cancer and miR-3117 was recently confirmed (Neerincx et al., 2015). The studies showed that overexpression of *miR-3117* could promote cell proliferation in HCC (Cui et al., 2017). Few studies have been done about researched miR-3117 and susceptibility to cancer. No study has reported the role of *miR-3117 *rs4655646 and rs7512692 polymorphisms in cancer. Our investigations on *miR-3117* rs4655646 polymorphism showed that the AG genotype, as well as G allele, increased the risk of BC in the sample of Iranian women. Similarly, our findings suggest that* miR-3117 *rs7512692 variant increased the risk of BC in CT and TT genotypes as well as T allele.

In conclusion, our findings suggest that miR-1307 rs7911488 and *miR-1269* rs73239138 polymorphisms reduced the risk of BC in women within the southeast region of Iran. However,* miR-3117* rs4655646 and *miR-3117* rs7512692 variants increased the risk of developing BC in the sample of Iranian women. Our findings could help scientists better understand the pathways that cause the BC development of BC and possible new methods and biomarkers to predict and fight this deadly disease. 

## References

[B1] Bao M, Song Y, Xia J (2018). miR-1269 promotes cell survival and proliferation by targeting tp53 and caspase-9 in lung cancer. Onco Targets Ther.

[B2] Bertoni N, de Souza MC, Crocamo S, Szklo M, de Almeida LM (2019). Is a family history of the breast cancer related to women’s cancer prevention behaviors?. Int J Behav Med.

[B3] Bhan A, Mandal SS (2015). LncRNA HOTAIR: A master regulator of chromatin dynamics and cancer. Biochim Biophys Acta.

[B4] Bray F, Ferlay J, Soerjomataram I (2018). Global cancer statistics 2018: GLOBOCAN estimates of incidence and mortality worldwide for 36 cancers in 185 countries. CA Cancer J Clin.

[B5] Bu P, Wang L, Chen K-Y, Rakhilin N (2015). miR-1269 promotes metastasis and forms a positive feedback loop with TGF-β. Nat Commun.

[B6] Bu P, Wang L, Chen K-Y (2015). miR-1269 promotes metastasis and forms a positive feedback loop with TGF-β. Nat Commun.

[B7] Bueno MJ, de Castro IP, Malumbres M (2008). Control of cell proliferation pathways by microRNAs. Cell Cycle.

[B8] Cui X, Li Q, He Y (2017). miR-3117 regulates hepatocellular carcinoma cell proliferation by targeting PHLPPL. Mol cell Biochem.

[B9] Dall GV, Britt KL (2017). Estrogen effects on the mammary gland in early and late life and breast cancer risk. Front Oncol.

[B10] Danesh H, Hashemi M, Bizhani F, Hashemi SM, Bahari G (2018). Association study of miR-100, miR-124-1, miR-218-2, miR-301b, miR-605, and miR-4293 polymorphisms and the risk of breast cancer in a sample of Iranian population. Gene.

[B11] Eckhardt BL, Francis PA, Parker BS, Anderson RL (2012). “Strategies for the discovery and development of therapies for metastatic breast cancer. Nat Rev Drug Discov.

[B12] Erdmann VA, Szymanski M, Hochberg A, Groot Nd, Barciszewski J (2000). Non-coding, mRNA-like RNAs database Y2K. Nucleic Acids Res.

[B13] Filipowicz W, Bhattacharyya SN, Sonenberg N (2008). “Mechanisms of post-transcriptional regulation by microRNAs: are the answers in sight?. Nat Rev Genet.

[B14] Gan T-Q, Tang R-X, He R-Q (2015). Upregulated MiR-1269 in hepatocellular carcinoma and its clinical significance. Int J Clin Exp Med.

[B15] García-Donas J, Beuselinck B, Inglada-Pérez L (2016). Deep sequencing reveals microRNAs predictive of antiangiogenic drug response. JCI Insight.

[B16] Gutierrez-Camino A, Martin-Guerrero I, Dolzan V (2018). “Involvement of SNPs in miR-3117 and miR-3689d2 in childhood acute lymphoblastic leukemia risk. Oncotarget.

[B17] Han S, Zou H, Lee J-W (2019). miR-1307-3p stimulates breast Cancer development and progression by targeting SMYD4. J Cancer.

[B18] Hashemi M, Bahari G, Tabasi F, Moazeni-Roodi A, Ghavami S (2018). Association between rs1862513 and rs3745367 genetic polymorphisms of resistin and risk of cancer: a meta-analysis. Asian Pac J Cancer Prev.

[B19] Hashemi M, Bojd HH, Nasab EE (2013). Association of adiponectin rs1501299 and rs266729 gene polymorphisms with nonalcoholic fatty liver disease. Hepat Mon.

[B20] Hashemi M, Sanaei S, Hashemi SM, Eskandari E, Bahari G (2018). Association of single nucleotide polymorphisms of the MDM4 gene with the susceptibility to breast cancer in a Southeast Iranian population sample. Clin Breast Cancer.

[B21] Hassanzarei S, Hashemi M, Sattarifard H (2017). Genetic polymorphisms of HOTAIR gene are associated with the risk of breast cancer in a sample of southeast Iranian population. Tumor Biol.

[B22] Hu Z, Chen J, Tian T (2008). “Genetic variants of miRNA sequences and non–small cell lung cancer survival. J Clin Invest.

[B23] Jayasekara H, MacInnis RJ, Hodge AM (2016). Is breast cancer risk associated with alcohol intake before first full-term pregnancy?. Cancer Causes Control.

[B24] Karami S, Sattarifard H, Kiumarsi M (2020). Evaluating the possible association between PD-1 (Rs11568821, Rs2227981, Rs2227982) and PD-L1 (Rs4143815, Rs2890658) polymorphisms and susceptibility to breast cancer in a sample of southeast Iranian women. Asian Pac J Cancer Prev.

[B26] Li W, Zhang H, Min P (2017). Downregulated miRNA1269a variant (rs73239138) decreases the susceptibility to gastric cancer via targeting ZNF70. Oncol Lett.

[B27] Liu F, Dear K, Huang L (2016). Association between microRNA-27a rs895819 polymorphism and risk of colorectal cancer: A meta-analysis. Cancer Genet.

[B28] McTiernan A, Friedenreich CM, Katzmarzyk PT (2019). Physical activity in cancer prevention and survival: A Systematic Review. Med Sci Sports Exerc.

[B29] Meijer H, Kong Y, Lu W (2013). Translational repression and eIF4A2 activity are critical for microRNA-mediated gene regulation. Science.

[B30] Min P, Li W, Zeng D (2017). A single nucleotide variant in microRNA-1269a promotes the occurrence and process of hepatocellular carcinoma by targeting to oncogenes SPATS2L and LRP6. Bull du Cancer.

[B31] Moazeni-Roodi A, Ghavami S, Hashemi M (2019). Association between miR-423 rs6505162 polymorphism and susceptibility to cancer: A Meta-Analysis. Arch Med Res.

[B32] Moazeni-Roodi A, Ghavami S, Hashemi M (2019). Lack of association between miR-605 rs2043556 polymorphism and overall cancer risk: a meta-analysis of case-control studies. MicroRNA.

[B33] Nazarian A, Mohamadnia A, Danaee E, Bahrami N (2019). Examining the expression of miR-205 and CEA mRNA in peripheral blood of patients with OSCC (Oral Squamous Cell Carcinomas) and comparing them with healthy people. Asian Pac J Cancer Biol.

[B34] Neerincx M, Sie D, Van De Wiel M (2015). MiR expression profiles of paired primary colorectal cancer and metastases by next-generation sequencing. Oncogenesis.

[B35] Neerincx M, Sie D, Van De Wiel M (2015). MiR expression profiles of paired primary colorectal cancer and metastases by next-generation sequencing. Oncogenesis.

[B36] Omrani M, Hashemi M, Eskandari-Nasab E (2014). hsa-mir-499 rs3746444 gene polymorphism is associated with susceptibility to breast cancer in an Iranian population. Biomarkers Med.

[B37] Payehghadr S, Bahrami N, Mohammadi F, Naji T, Mohamadnia A (2018). Detection of miR-21, MUC1mRNA and VEGF protein biomarkers expression changes in oral squamous cell carcinomas (OSCC) in peripheral blood. Asian Pac J Cancer Biol.

[B38] Peng Y, Croce CM (2016). The role of MicroRNAs in human cancer. Signal Transduct Target Ther.

[B39] Pizot C, Boniol M, Mullie P (2016). Physical activity, hormone replacement therapy and breast cancer risk: A meta-analysis of prospective studies. Eur J Cancer.

[B40] Qi P, Wang L, Zhou B (2015). Associations of miRNA polymorphisms and expression levels with breast cancer risk in the Chinese population. Genet Mol Res.

[B41] Qiu X, Dou Y (2017). miR-1307 promotes the proliferation of prostate cancer by targeting FOXO3A. Biomed Pharmacotherapy.

[B42] Rafighdoost F, Rafighdoost A, Rafighdoost H (2015). The 19-bp deletion polymorphism of dihydrofolate reductase (DHFR) and nonsyndromic cleft lip with or without cleft palate: evidence for a protective role. J Appl Oral Sci.

[B43] Rafighdoost H, Hashemi M, Asadi H, Bahari G (2018). “Association of single nucleotide polymorphisms in WNT genes with the risk of nonsyndromic cleft lip with or without cleft palate. Congenital Anomalies.

[B44] Rafighdoost H, Hashemi M, Danesh H (2017). Association of single nucleotide polymorphisms in AXIN2, BMP4, and IRF6 with Non-Syndromic Cleft Lip with or without Cleft Palate in a sample of the southeast Iranian population. J Appl Oral Sci.

[B45] Rafighdoost H, Tabatabaei F, Bahari G, Hashemi M (2019). “Association of single nucleotide polymorphisms in TPM1 rs11071720, rs3803499, rs12148828, and rs1972041 with the risk of nonsyndromic cleft lip with or without cleft palate in a sample of the Iranian population, a preliminary report. Ann Hum Genet.

[B46] Rinn JL, Chang HY (2012). Genome regulation by long noncoding RNAs. Ann Rev Biochem.

[B47] Sadeghi-Bojd S, Falsafinejad F, Danesh H (2019). Macrophage migration inhibitory factor-173 G> C gene polymorphism is associated with increased risk of XNephrotic syndrome in children. Iran J Kidney Dis.

[B48] Sattarifard H, Hashemi M, Hassanzarei S (2019). Long non-coding RNA POLR2E gene polymorphisms increased the risk of prostate cancer in a sample of the Iranian population. Nucleosides Nucleotides Nucleic Acids.

[B49] Shimomura A, Shiino S, Kawauchi J (2016). Novel combination of serum microRNA for detecting breast cancer in the early stage. Cancer Sci.

[B50] Shiyanbola OO, Arao RF, Miglioretti DL (2017). Emerging trends in family history of breast cancer and associated risk. Cancer Epidemiol Prev Biomarkers.

[B51] Sibin M, Harshitha S, Narasingarao K (2017). Effect of rs11614913 polymorphism on mature miR196a2 expression and its target gene HOXC8 expression in human glioma. J Mol Neuroscience.

[B52] Tang R, Qi Q, Wu R (2015). The polymorphic terminal-loop of pre-miR-1307 binding with MBNL1 contributes to colorectal carcinogenesis via interference with Dicer1 recruitment. Carcinogenesis.

[B53] Umami A, Sudalhar S, Pratama TWY, Fitri I, Firmansyah A (2020). Knowledge, barriers, and motivation related to breast and cervical cancer screening among women in Bojonegoro, East Java: A Qualitative Study. J Health Promot Behav.

[B54] Vickers NJ (2017). Animal communication: When i’m calling you, will you answer too?. Curr Biol.

[B55] Wang C, Li L, Yin Z (2016). An Indel Polymorphism within pre-miR3131 confers risk for hepatocellular carcinoma. Carcinogenesis.

[B56] Wang X, Zhu J (2018). Mir-1307 regulates cisplatin resistance by targeting Mdm4 in breast cancer expressing wild type P53. Thoracic Cancer.

[B58] Wu J, Wang Y, Shang L, Qi L, Song M (2018). Five common functional polymorphisms in microRNAs and susceptibility to breast cancer: An Updated Meta-Analysis. Genet Testing Mol Biomarkers.

[B59] Xiong G, Wang Y, Ding Q, Yang L (2015). Hsa-mir-1269 genetic variant contributes to hepatocellular carcinoma susceptibility through affecting SOX6. Am J Translat Res.

[B60] Yang X-W, Shen G-Z, Cao L-Q (2014). MicroRNA-1269 promotes proliferation in human hepatocellular carcinoma via downregulation of FOXO1. BMC Cancer.

[B61] Yang Z, Li R, Ao J (2018). miR-1307-3p suppresses the chondrogenic differentiation of human adipose-derived stem cells by targeting BMPR2. Int J Mol Med.

[B62] Yu D (2019). The impact of exercise during radiation therapy for breast cancer patients. SAGE Open Med.

[B63] Zhou Y, Wang M, Wu J (2015). The clinicopathological significance of miR-1307 in chemotherapy resistant epithelial ovarian cancer. J Ovarian Res.

